# Toxicity of long chain fatty acids towards acetate conversion by *Methanosaeta concilii* and *Methanosarcina mazei*

**DOI:** 10.1111/1751-7915.12365

**Published:** 2016-06-08

**Authors:** Sérgio A. Silva, Andreia F. Salvador, Ana J. Cavaleiro, M. Alcina Pereira, Alfons J. M. Stams, M. Madalena Alves, Diana Z. Sousa

**Affiliations:** ^1^ Centre of Biological EngineeringUniversity of MinhoBragaPortugal; ^2^ Laboratory of MicrobiologyWageningen UniversityWageningenThe Netherlands

## Abstract

Long‐chain fatty acids (LCFA) can inhibit methane production by methanogenic archaea. The effect of oleate and palmitate on pure cultures of *Methanosaeta concilii* and *Methanosarcina mazei* was assessed by comparing methane production rates from acetate before and after LCFA addition. For both methanogens, a sharp decrease in methane production (> 50%) was observed at 0.5 mmol L^−1^ oleate, and no methane was formed at concentrations higher than 2 mmol L^−1^ oleate. Palmitate was less inhibitory than oleate, and *M. concilii* was more tolerant to palmitate than *M. mazei,* with 2 mmol L^−1^ palmitate causing 11% and 64% methanogenic inhibition respectively. This study indicates that *M. concilii* and *M. mazei* tolerate LCFA concentrations similar to those previously described for hydrogenotrophic methanogens. In particular, the robustness of *M. concilii* might contribute to the observed prevalence of *Methanosaeta* species in anaerobic bioreactors used to treat LCFA‐rich wastewater.

## Introduction

Long‐chain fatty acids (LCFA) are released during lipid hydrolysis, and hold the majority of the energy potential of these biomolecules (Alves *et al*., 2009). LCFA are degraded by anaerobic bacteria through β‐oxidation to form acetate and hydrogen, which are then used by acetoclastic and hydrogenotrophic methanogens to produce methane. In anaerobic bioreactors, approximately 70% of the methane produced from LCFA results from acetoclastic activity, whereas about 30% derives from hydrogenotrophic activity (Sousa *et al*., 2009). Low methane production has been reported during continuous bioreactor operation with LCFA, which was associated with inhibition and toxicity of these compounds towards the methanogenic communities (Chen *et al*., 2014; Dereli *et al*., 2014).

Studies on the toxic effect of LCFA on the acetoclastic and hydrogenotrophic activities of anaerobic sludge indicate that acetoclastic methanogens are the most sensitive to LCFA (Alves *et al*., 2009; Palatsi *et al*., 2010). Nevertheless, results obtained in our research group showed a significant increase in the relative abundance of methanogens in anaerobic sludge exposed to continuous feeding of oleate (C_18:1_) and palmitate (C_16:0_) followed by batch incubation (Sousa *et al*., 2007). Endurance of acetoclastic methanogens in a continuous bioreactor treating LCFA‐rich effluent, at organic loading rates up to 21 kg m^−3^ day^−1^, has also been reported (Salvador *et al*., 2013). Additionally, activity of acetoclastic methanogens in sludge incubated with LCFA in batch assays has been shown, as more than 80% of the proteins assigned to the archaeal community were from *Methanosaeta concilii* (Salvador, 2013). The prevalence of acetoclastic methanogens belonging to *Methanosaeta* and *Methanosarcina* genera in LCFA‐degrading environments has been reported in other studies (Shigematsu *et al*., 2006; Palatsi *et al*., 2010; Baserba *et al*., 2012; Ma *et al*., 2015), suggesting some controversy in the reported sensitivity of acetoclastic methanogens to LCFA.

In lipid‐containing wastewaters, oleate (C_18:1_) is generally the most abundant LCFA, and palmitate (C_16:0_) tends to accumulate in anaerobic bioreactors treating these effluents (Pereira *et al*., 2002; Dereli *et al*., 2014). The effect of LCFA on anaerobic sludge has been studied before (Sousa *et al*., 2007; Palatsi *et al*., 2010; Silva *et al*., 2014), and a few studies report the sensitivity of pure cultures of hydrogenotrophic methanogens (Sousa *et al*., 2013; Zhou *et al*., 2013). Information on the sensitivity of pure cultures of acetoclastic methanogens is lacking. *Methanosaeta concilii* and *Methanosarcina mazei* were the ones commonly found in mesophilic anaerobic bioreactors treating LCFA‐based wastewaters (Table S1). In this work, the effect of saturated (C_16:0_, palmitate) and unsaturated (C_18:1_, oleate) LCFA on the acetoclastic methanogenesis of pure cultures of *M. concilii* and *M. mazei* was investigated.

## Results and discussion

**Figure 1:**
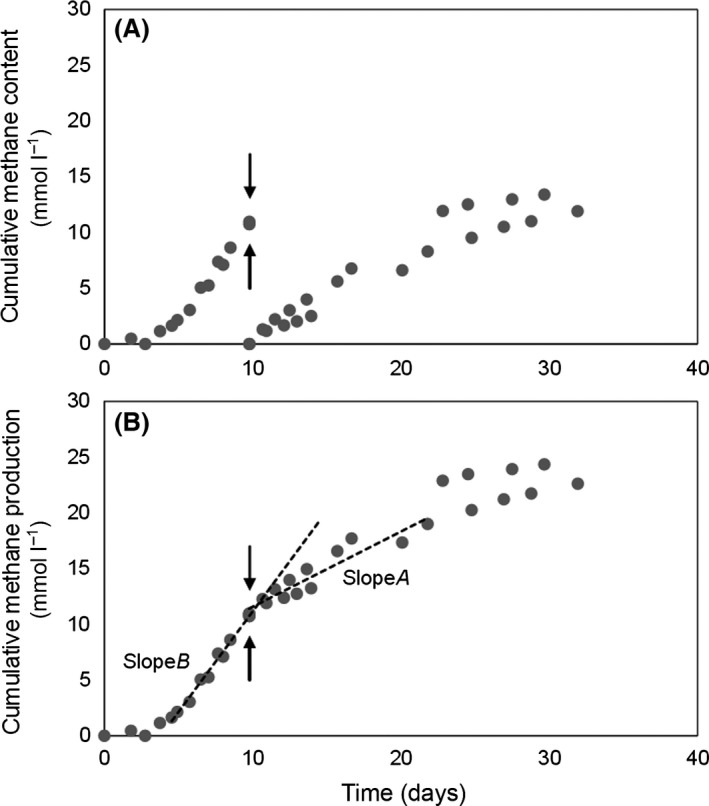
Cumulative methane production from acetate by *M. mazei* before and after the addition of 0.5 mmol L^−1^ of oleate: (A) cumulative methane content measured in the bottles headspace; (B) cumulative methane production mathematically adjusted. Dashed lines represent the methane production rate (mmol L^−1^ day^−1^) before (Slope*B*) and after (Slope*A*) LCFA addition. Arrow points the moment of headspace flushing and second acetate addition (↑) and LCFA addition (↓). *M. mazei* (DSM 2053^T^) was acquired from the Deutsche Sammlung von Mikroorganismen und Zellkulturen (DSMZ, Germany) and was grown under strict anaerobic conditions as described by Stams *et al*. (1993) with sodium acetate as substrate at a final concentration of 20 mmol L^−1^. Methane production was quantified over time until the mid‐exponential phase was achieved. At this point, the methane accumulated in the headspace was quantified by gas chromatography, and after was removed under sterile conditions by flushing with 80% N_2_ and 20% CO_2_ gas mixture. To avoid substrate limitation during the exposure to LCFA, 10 mmol L^−1^ of acetate was added at the moment of LCFA addition. Assays were performed in duplicate and bottles were incubated in the dark at 37°C, without agitation.

*Methanosaeta concilii* (DSM 3671^T^) and *M. mazei* (DSM 2053^T^) were grown on sodium acetate as substrate for methane production, which was quantified over time before and after LCFA addition (Figs S1 and S2). Differences in methane production rate before and after LCFA addition were used to determine the methanogenic inhibition at oleate or palmitate concentrations of 0.5, 1, 2 and 4 mmol L^−1^ (see Fig. 1 as example).

Slope ratio (*S*_ratio_) was calculated for each incubation condition according to equation *S*_ratio_ = Slope*A*/Slope*B*, where Slope*B* and slope*A* represent the cumulative methane production slopes (mmol L^−1^ day^−1^) before and after the headspace flushing, second acetate addition and LCFA (oleate or palmitate) addition. Methane production rate of *M. concilii* was affected by oleate, as shown by the sharp decrease in the *S*_ratio_ with 0.5 mmol L^−1^ oleate, compared with the *S*_ratio_ obtained in the control (Table 1). This corresponds to 67% methanogenic inhibition and thus the IC_50_ of oleate was below 0.5 mmol L^−1^. No methane was produced, nor was acetate consumed, after the addition of 2 and 4 mmol L^−1^ oleate to *M. concilii* cultures (Table 1 and Table S2), which suggests complete methanogenic inhibition. Palmitate also affected methane production of *M. concilii*, although not as much as oleate, i.e. palmitate concentrations up to 2 mmol L^−1^ resulted in a maximum inhibition of 11%, whereas in the presence of 4 mmol L^−1^ methanogenesis was inhibited by 94% (Table 1).

**Table 1:** Slopes ratio (*S*_ratio_) calculated for *Methanosaeta concilii* and *Methanosarcina mazei* in the presence of different oleate and palmitate concentrations

	LCFA/mmol L^−1^	Slope*B*	Slope*A*	*S*_ratio_ a ^,^ c	Inhibition/%^b^^,^ c
*M. concilii*					
	Control	1.1 ± 0.1	1.9 ± 0.1	1.8 ± 0.2	–
Oleate	0.5	1.1 ± 0.1	0.6 ± 0.2	0.6 ± 0.2	67 ± 13
	1	1.1 ± 0.1	0.3 ± 0.0	0.3 ± 0.0	83 ± 14
	2	1.0 ± 0.1	0.0 ± 0.0	0.0 ± 0.0	100 ± 16
	4	1.0 ± 0.1	0.0 ± 0.1	0.0 ± 0.1	100 ± 16
Palmitate	0.5	1.1 ± 0.1	1.9 ± 0.1	1.8 ± 0.2	0
	1	1.1 ± 0.1	1.8 ± 0.3	1.7 ± 0.3	6 ± 22
	2	1.1 ± 0.1	1.8 ± 0.1	1.6 ± 0.1	11 ± 11
	4	1.2 ± 0.1	0.1 ± 0.1	0.1 ± 0.1	94 ± 15
*M. mazei*					
	Control	2.2 ± 0.2	2.4 ± 0.1	1.1 ± 0.1	–
Oleate	0.5	1.8 ± 0.1	0.7 ± 0.1	0.4 ± 0.0	64 ± 11
	1	1.1 ± 0.2	0.4 ± 0.1	0.4 ± 0.1	64 ± 19
	2	1.8 ± 0.1	0.1 ± 0.0	0.0 ± 0.0	100 ± 14
	4	1.7 ± 0.2	0.1 ± 0.1	0.0 ± 0.1	100 ± 14
Palmitate	0.5	1.5 ± 0.2	1.7 ± 0.2	1.1 ± 0.2	0
	1	1.2 ± 0.2	1.5 ± 0.1	1.2 ± 0.2	0
	2	2.1 ± 0.2	0.9 ± 0.2	0.4 ± 0.1	64 ± 22
	4	2.0 ± 0.6	0.1 ± 0.2	0.0 ± 0.1	100 ± 14

a Differences in methane production rate before and after LCFA addition were expressed as a slope ratio (*S*_ratio_) that was calculated for each condition using the cumulative methane production slope, according to the equation (*S*_ratio_ = Slope*A*/Slope*B*). For control assays, in which no LCFA was added, *S*_ratio_ were equally calculated and *SlopeA* determined after the headspace flushing and second acetate addition (Figs S1 and S2).

b The inhibitory effect of the different LCFA concentrations on methane production was expressed in percentage, by comparing the *S*_*ratio*_ obtained from the LCFA supplemented assays (*S*_ratio_*L*_) with the slopes ratio obtained from the control assays (*S*_ratio_*C*_), according to equation (Inhibition = ((*S*_ratio_*C*_ − *S*_ratio_*L*_)/*S*_ratio_*C*_)*100).

c Average ± standard deviation of duplicate assays.

Similar results were obtained with *M. mazei* in the presence of oleate, which caused 64% inhibition at 0.5 mmol L^−1^ and complete inhibition at 2 and 4 mmol L^−1^ (Table 1 and Table S2). However, although *Methanosarcina* spp. are reported as highly tolerant to others toxicants, such as ammonia and salts (De Vrieze *et al*., 2012; Hao *et al*., 2015), *M. mazei* was more vulnerable to palmitate than *M. concilii*. Palmitate concentrations of 2 mmol L^−1^ caused a 64% decrease of methane production by *M. mazei*, while for the same concentration the methanogenic inhibition of *M. concilii* was only 11% (Table 1).

The predominance of *Methanosaeta* spp. in anaerobic reactors containing high concentrations of palmitate (Shigematsu *et al*., 2006; Salvador *et al*., 2013) also indicates that *Methanosaeta* spp. might be more tolerant than *Methanosarcina* spp. In methanogenic bioreactors treating LCFA‐based wastewater, the presence of both *Methanosaeta* and *Methanosarcina* species has been reported. *Methanosaeta* spp. are usually the dominant acetoclastic methanogens when acetate concentrations are low, due to their higher affinity for acetate compared with *Methanosarcina* spp., while *Methanosarcina* spp are generally more abundant at high acetate concentrations (De Vrieze *et al*., 2012). Nevertheless, *Methanosaeta* was found to persist and dominate over *Methanosarcina* in unstable anaerobic bioreactors with acetate concentrations up to 44 mmol L^−1^ (Chen and He, 2015). The prevalence of these acetoclastic microorganisms in LCFA‐degrading environments might also be influenced by their different sensitivity to these compounds.

A comparison between IC_50_ values obtained for oleate and palmitate towards acetoclastic methanogens in this study, and towards hydrogenotrophic methanogens (Sousa *et al*., 2013) is presented in Table 2. Our results show that *M. concilii* and *M. mazei* are similarly affected by the presence of oleate as the hydrogenotroph *Methanospirillum hungatei,* and *M. concilii* seems to be even more tolerant to the presence of palmitate than *M. hungatei*. Previous studies on the toxicity of LCFA towards anaerobic sludge highlighted the higher sensitivity of acetoclasts compared with hydrogenotrophs. Since LCFA can absorb to the cells at variable amounts, its toxicity might be explained by a physical inhibition phenomenon rather than by direct metabolic inhibition (Pereira *et al*., 2005). Mass transfer limitations exerted by LCFA are likely more pronounced for acetate than for hydrogen transport, since hydrogen is a smaller molecule (Pereira *et al*., 2005).

**Table 2:** Concentration (mmol L^−1^) of oleate and palmitate necessary to inhibit in 50% methanogenesis of pure cultures of acetoclastic and hydrogenotrophic methanogens

LCFA	Acetoclastic methanogens		Hydrogenotrophic methanogens^a^	
	*Methanosaeta concilii*	*Methanosarcina mazei*	*Methanospirillum hungatei*	*Methanobacterium formicicum*
Oleate	< 0.5	< 0.5	< 0.5	< 1
Palmitate	]2–4[	]1–2]	]1–2[	> 4

a Sousa *et al*. (2013).

Differences in cell envelopes composition might also influence the sensitivity of microorganisms. For example, the cell wall of *Methanosarcina* contains methanochondroitin and the one of *Methanosaeta* contains a sheath surrounding the S‐layer and the cytoplasmic membranes. The sheath might have a protective effect since it is reported to be resistant to detergents (Claus and König, 2010).

Although the studies with mixed communities degrading LCFA are important, information about the sensitivity of individual species growing in pure cultures show the unequivocal metabolic behaviour of each tested species in the presence of LCFA.

The tolerance to LCFA can be higher when methanogens are growing in complex microbial communities than in pure cultures, due to a structural protection provided by aggregation of different microbial species. IC_50_ values between 0.1 and 1 mmol L^−1^ and approximately 3 mmol L^−1^ were reported for suspended and granular sludge respectively (Table S3). These values are, however, close to the ones obtained in this study for acetoclastic methanogens, and by Sousa *et al*. (2013) for pure cultures of hydrogenotrophic methanogens, and are indicative of LCFA concentrations that might cause operational problems due to direct inhibition of methanogens. These studies, all together, allow to discriminate between mass transport‐related physical inhibition and metabolic inhibition, which impacts practical applications of anaerobic processes for treatment of LCFA‐containing wastewater.

Because in continuous bioreactors oleate is for a large part converted to palmitate (Pereira *et al*., 2002), the potential toxicity of palmitate is, in this context, most relevant. Palmitate concentrations between 1 and 2 mmol L^−1^ can be tolerated by methanogenic communities, allowing to feed higher oleate concentrations than the IC_50_ for oleate (<0.5 mmol L^−1^). The higher IC_50_ exhibited by the acetoclastic *M. concilii* and the hydrogenotroph *Methanobacterium formicicum* (Table 2), particularly for palmitate, may explain why these species are commonly found in bioreactors and point to their importance in the conversion of LCFA to methane in anaerobic wastewater treatment systems.

## Conclusions

In this work, two acetoclastic methanogens revealed different tolerance to LCFA. *Methanosaeta concilii* demonstrated a tolerance to palmitate similar to hydrogenotrophic methanogens, which generally are considered to be more resistant. These results are relevant in the context of lipid‐rich wastewater treatment, where the presence and prevalence of *Methanosaeta* species could be a good indicator of the system potential to efficiently convert LCFA to methane.

## Conflict of Interest

The authors declare no conflict of interest.

## Supporting information


**Fig. S1.** Cumulative methane production from acetate consumption by *Methanosaeta concilii* during exposure to oleate or palmitate.
**Fig. S2.** Cumulative methane production from acetate consumption by *Methanosarcina mazei* during exposure to oleate or palmitate.
**Table S1.** Main acetoclastic methanogens detected in anaerobic sludges from LCFA‐fed reactors.
**Table S2.** Acetate concentration (mmol L^−1^) determined before LCFA was added (Ac_init_) and at the end of the assays (Ac_end_).
**Table S3.** Inhibition of acetoclastic methanogenic activity by LCFA, in several sludges exposed to different wastewater compositions.
