# Supplementary material for: Toxicity of long chain fatty acids towards acetate conversion by Methanosaeta concilii and Methanosarcina mazei
Source: Microb Biotechnol. 2016 Jun 8;9(4):514–8. doi: 10.1111/1751-7915.12365 (PMC4919993; doi:10.1111/1751-7915.12365)
Supplement: Supplementary file 1 — Fig. S1. Cumulative methane production from acetate consumption by Methanosaeta concilii during exposure to oleate or palmitate. Fig. S2. Cumulative methane production from acetate consumption by Methanosarcina mazei during exposure to oleate or palmitate. Table S1. Main acetoclastic methanogens detected in anaerobic sludges from LCFA‐fed reactors. Table S2. Acetate concentration (mmol L−1) determined before LCFA was added (Acinit) and at the end of the assays (Acend). Table S3. Inhibition of acetoclastic methanogenic activity by LCFA, in several sludges exposed to different wastewater compositions. [file MBT2-9-514-s001.docx]

**Supporting Information**

Toxicity of long chain fatty acids towards acetate conversion by *Methanosaeta concilii* and *Methanosarcina mazei*

Sérgio A. Silva,^a^ Andreia F. Salvador,^a^ Ana J. Cavaleiro,^a^ M. Alcina Pereira,^a^ Alfons J. M. Stams,^a,b^ M. Madalena Alves,^a^ Diana Z. Sousa,^a,b^

^a^ Centre of Biological Engineering, University of Minho, Braga, Portugal.

^b^ Laboratory of Microbiology, Wageningen University, Wageningen, The Netherlands

Corresponding Author:

Ana J. Cavaleiro

Address: Departamento de Engenharia Biológica, Universidade do Minho, Campus de Gualtar, 4710-057 Braga, Portugal

Email: acavaleiro@deb.uminho.pt

Telephone: 00351253604423

Fax: 00351253604429

**Fig. S1** – Cumulative methane production from acetate consumption by *Methanosaeta concilii* (DSM 3671^T^) during exposure to oleate or palmitate. Controls a) were performed without LCFA addition. Assays performed with oleate: b) 0.5 mmol L^-1^; c) 1 mmol L^-1^; d) 2 mmol L^-1^; e) 4 mmol L^-1^. Assays performed with palmitate: f) 0.5 mmol L^-1^; g) 1 mmol L^-1^; h) 2 mmol L^-1^; i) 4 mmol L^-1^. Dashed line and whole line represent the slope before and after LCFA addition, respectively. Arrows represent the moment of headspace flushing and second acetate addition (↑), and LCFA addition (↓). Samples from the gas phase were collected over time for methane quantification by gas chromatography (GC) as described elsewhere (Silva *et al*., 2014).

**Fig. S2** – Cumulative methane production from acetate consumption by *Methanosarcina mazei* during exposure to oleate or palmitate. Controls a) were performed without LCFA addition. Assays performed with oleate: b) 0.5 mmol L^-1^; c) 1 mmol L^-1^; d) 2 mmol L^-1^; e) 4 mmol L^-1^. Assays performed with palmitate: f) 0.5 mmol L^-1^; g) 1 mmol L^-1^; h) 2 mmol L^-1^; i) 4 mmol L^-1^. Dashed line and whole line represent the slope before and after LCFA addition, respectively. Arrows represent the moment of headspace flushing and second acetate addition (↑), and LCFA addition (↓).Samples from the gas phase were collected over time for methane quantification by gas chromatography (GC) as described elsewhere (Silva *et al*., 2014).

**Table S1** – Main acetoclastic methanogens detected in anaerobic sludges from LCFA-fed reactors.

| **References** | **LCFA** | **OLR/** | **Conditions** | **Main Acetoclasts** |
| --- | --- | --- | --- | --- |
| Pereira *et al*. 2002^*^ | Oleate | Up to 8 kg m^-3^ d^-1^ | Continuous | *Methanosaeta concilii* |
| Shigematsu *et al*. 2006 | Oleate/Palmitate | 1 kg m^-3^ d^-1^ | Continuous | *Methanosaeta concilii* |
| Sousa *et al*. 2007 | Oleate/Palmitate | 4 kg m^-3^ d^-1^ | Continuous  Batch | *Methanosaeta concilii*  *Methanosarcina*  *mazei* |
| Salvador *et al*. 2013 | Oleate | Up to 21 kg m^-3^ d^-1^ | Step-feeding | *Methanosaeta concilii* |

^*^Pereira *et al*. (2002) Molecular monitoring of microbial diversity in expanded granular sludge bed (EGSB) reactors treating oleic acid. *FEMS Microbiol Ecol* **41**: 95 – 103

**Table S2** – Acetate concentration (mmol L^-1^) determined before LCFA was added (Ac_init_) and at the end of the assays (Ac_end_).

|  |  | ***Methanosaeta concilii*** | | ***Methanosarcina mazei*** | |
| --- | --- | --- | --- | --- | --- |
| **Assay** | **LCFA/**  **mmol L^-1^** | **Ac_init_^a^**  **mmol L^-1^** | **Ac_end_^a^**  **mmol L^-1^** | **Ac_init_^a^**  **mmol L^-1^** | **Ac_end_^a^**  **mmol L^-1^** |
| Control | - | 12.4 ± 0.7 | n.d. | 14.2 ± 0.7 | n.d. |
| Oleate | 0.5 | 16.0 ± 2.6 | n.d. | 14.5 ± 2.1 | 1.7 ± 1.1 |
|  | 1 | 13.6 ± 0.0 | n.d. | 14.8 ± 2.7 | 5.7 ± 0.5 |
|  | 2 | 14.6 ± 0.0 | 14.2 ± 0.2 | 17.2 ± 1.3 | 16.4 ± 1.2 |
|  | 4 | 13.2 ± 0.3 | 12.0 ± 0.3 | 17.3 ± 0.4 | 16.0 ± 0.8 |
| Palmitate | 0.5 | 13.1 ± 0.0 | n.d. | 14.1 ± 2.6 | 1.5 ± 0.1 |
|  | 1 | 13.1 ± 0.6 | n.d. | 16.4 ± 1.5 | n.d. |
|  | 2 | 13.3 ± 0.2 | n.d. | 15.0 ± 0.0 | 2.0 ± 1.2 |
|  | 4 | 13.0 ± 0.7 | 12.6 ± 0.2 | 16.5 ± 0.8 | 17.8 ± 0.6 |

Liquid samples were collected before adding the LCFA (Ac_init_) and at the end of the toxicity assays (Ac_end_) for acetate quantification. Acetate was analysed by high-performance liquid chromatography (HPLC) (Silva *et al*., 2014). LCFA – Long chain fatty acid. **n.d.** – Not detected.

**a.** Average ± standard deviation of duplicate assays.

**Table S3** – Inhibition of acetoclastic methanogenic activity by LCFA, in several sludges exposed to different wastewater compositions

| **LCFA** | **IC_50_/**  **mmol L^-1^** | **Type of Sludge** | **Sludge previously exposed to:** | **Reference** |
| --- | --- | --- | --- | --- |
| Oleate | 0.16 – 0.23 | Suspended | Whole milk | ^1^ |
|  | 0.13 – 0.97 | Suspended | Skim milk | ^1^ |
|  | 0.28 – 0.45 | Suspended | Oleate + skim milk | ^1^ |
|  | 0.10 – 0.13 | Suspended | Oleate | ^1^ |
|  | 3.55 | Granular | LCFA mixture + Glucose | ^2^ |
|  | 3.10 | Granular | Dairy wastewater | ^3^ |
|  | 4.35 | Granular | Potato processing wastewater | ^4^ |
|  | 0.25 | Suspended | Skim milk + oleate | ^5^ |
| Palmitate | 4.32 | Suspended | Skim milk + oleate | ^5^ |

^1^ Alves *et al*. (2001) Effects of lipids and oleic acid on biomass development in anaerobic fixed-bed reactors Part II: Oleic acid toxicity and biodegradability. *Water Res* **35**: 264-270.

^2^ Shin *et al*. (2003) Inhibitory effects of long-chain fatty acids on VFA degradation and β-oxidation. *Water Sci Technol* **47**: 139-146.

^3^ Kim *et al*. (2004) Kinetics of LCFA inhibition on acetoclastic methanogenesis, propionate degradation and β-oxidation. *J Environ Sci Heal A* **A39**: 1025-1037.

^4^ Koster and Cramer (1987) Inhibition of methanogenesis from acetate in granular sludge by long-chain fatty acids. *Appl Environ Microb* **53**: 403-409.

^5^ Pereira *et al*. (2005) Anaerobic biodegradation of oleic and palmitic acids: evidence of mass transfer limitations caused by long chain fatty acid accumulation onto the anaerobic sludge. *Biotechnol Bioeng* **92**: 15-23.
